# Malaria treatment perceptions, practices and influences on provider behaviour: comparing hospitals and non-hospitals in south-east Nigeria

**DOI:** 10.1186/1475-2875-8-246

**Published:** 2009-10-28

**Authors:** Obinna Onwujekwe, Benjamin Uzochukwu, Nkem Dike, Nkoli Uguru, Emmanuel Nwobi, Elvis Shu

**Affiliations:** 1 Department of Health Administration and Management, College of Medicine, University of Nigeria, Enugu, Nigeria; 2 Health Policy Research Group, Department of Pharmacology and Therapeutics, College of Medicine, University of Nigeria, Enugu, Nigeria; 3 Department of Community Medicine, College of Medicine, University of Nigeria, Enugu, Nigeria; 4 Roberta Buffett Center for International & Comparative Studies, Northwestern University, Chicago, USA; 5 Department of Preventive Dentistry, Faculty of Dentistry, College of Medicine, University of Nigeria, Enugu, Nigeria

## Abstract

**Background:**

People seek treatment for malaria from a wide range of providers ranging from itinerant drug sellers to hospitals. However, there are lots of problems with treatment provision. Hence, factors influencing treatment provision in hospitals and non-hospitals require further investigation in order to remedy the situation.

**Objectives:**

To examine the knowledge, pattern of treatment provision and factors influencing the behaviour of hospitals and non-hospitals in the treatment of malaria, so as to identify loci for interventions to improve treatment of the disease.

**Methods:**

A pre-tested structured questionnaire was used to collect data from 225 providers from hospitals and non-hospitals about their malaria treatment practices and factors that influence their provision of malaria treatment services in south-east Nigeria. The data from hospitals and other providers were compared for systematic differences.

**Results:**

73.5% of hospitals used microscopy to diagnose malaria and only 34.5.1% of non-hospitals did (p < 0.05). Majority of the respondents considered ability to pay bills (35.2%), already existing relationship (9.4%) and body mechanism (35.2%) of the patient before they provided malaria treatment services. Pressure from wholesalers to providers to repay the cost of supplied drugs was the major influence of the type of drugs provided to patients.

**Conclusion:**

There are many challenges to appropriate provision of malaria treatment services, although challenges are less in hospitals compared to other types of non-hospitals. Improving proper diagnosis of malaria and improving the knowledge of providers about malaria are interventions that could be used to improve malaria treatment provision.

## Background

Malaria is the major cause of morbidity and mortality in Nigeria, and particularly affects children under five years of age [1]. Worldwide, it kills more than one million people each year and between 20 and 40 percent of outpatient visits and between 10 and 15 percent of hospital admissions in Africa are attributed to malaria [2-4]. However, the prevailing method for diagnosing malaria in SSA is by clinical impression, which, in turn, typically amounts to treating all fevers as malaria [5]. Hence, there is a potential bias for overestimating the burden of the disease.

People seek treatment for malaria from a wide range of sources ranging from itinerant drug sellers to hospitals, but they often resort to the unregulated private commercial sector, where treatment may be inappropriate, although access costs may be lower [6]. Recourse to multiple providers is also common, and patients often begin with self-treatment using drugs purchased through the commercial sector, and then seek care from formal health providers [6,7]. If a patient is very ill, the public sector may be preferred because of the presence of more sophisticated equipment and a greater range of staff [8]. Patients may feel that private providers charge very high prices and are often skeptical about the motivation of private providers, believing them to be primarily interested in generating income for themselves rather than in the welfare of their patients [6,7,9].

However, there are many problems with treatment provision and existing practices, as well as factors influencing treatment provision require further investigation in order to remedy the situation. Over the years of deployment of anti-malarial drugs, there was little incentive to improve the way the drugs were used because chloroquine and sulphadoxine-pyrimethamine (SP) were inexpensive making it more cost-effective to treat presumptively rather than to get microscopic confirmation [5]. A previous study based on an audit of 665 patient records from public and private hospitals found that 45% of patients had diagnostic blood slides, 77% were prescribed monotherapies and only 3% were prescribed artemisin-based combination therapy (ACT) [10]. It has also been found for private shops that a large percentage of the drugs provided or dosages given, or both, are inappropriate, indicating the need for innovative and effective approaches to achieve rational prescribing practices [11].

There is paucity of knowledge about the level of knowledge of different providers about malaria, which will have strong influences on their malaria treatment practices. However, some researchers found that primary healthcare providers had poor knowledge about prevention strategies for malaria [12], and also that amongst primary healthcare providers, knowledge of some basic concepts of malaria was fairly adequate but treatment practices were poor [13]. Hence they recommended that there should be periodic education programmes, especially for health workers with many years of experience to help them maintain clinical skills and refresh their knowledge [13]. In a study, to determine patent medicine dealers' perspectives on malaria in south-east Nigeria, it was found that although the providers had fairly good knowledge about the causes and treatment of malaria, their treatment provision practices were sub-optimal [2]. However, some providers stated that malaria could be caused by the sun and drinking bad water [2]

There is also paucity of knowledge about the factors that influence the type of treatment that healthcare providers provide for the treatment of malaria and how the information can be used to improve treatment provision. The behaviour of providers is often influenced by their knowledge, financial incentives, competition, their perceptions of patients' attitudes, and any legal or regulatory sanctions for inappropriate behaviour [6]. It was also postulated that prescribing patterns are more likely to follow patient demands and expectations as well as profit motive rather than professional principles [2]. However, previous studies have reported that clinicians rarely perceived patient demand for anti-malarials and asserted that such demand for medication would not affect their prescribing behaviour [14].

It is rationally expected that provision of anti-malarial drugs would be rational in hospitals compared to other providers such as retailers like patent medicine dealers (PMDs) and other low level providers that may not have properly trained health personnel such as doctors. Inappropriate provision of drugs can lead to many untoward effects and decrease the effectiveness of treatment, as well as lead to poor adherence [10,15]. Some researchers found a wide variety of anti-malaria drug prescribing including chloroquine, SP and ACT, but with overall prescribing in government and private facilities being similar [10]. Few retailers understood their role in the delivery of primary health care for instance about 80.0% of the non-pharmacist - retailers considered themselves as traders rather than as healthcare providers [16]. Polypharmacy, where providers prescribe additional unnecessary drugs, is widespread [6,17]. In recent times, four primarily social, spheres of influence on malaria over-diagnosis were identified: firstly, the influence of initial training within a context where the importance of malaria is strongly promoted. Secondly, the influence of peers, conforming to perceived expectations from colleagues. Thirdly, pressure to conform with perceived patient preferences. Lastly, quality of diagnostic support, involving resource management, motivation and supervision [18].

The paper explores the reasonable uncharted ground of comparing hospitals with non-hospitals based on the premise that public and private hospitals are basically similar in terms of type of people that actually provide services there as well as in terms of malaria treatment practices. The paper does not tread the usual comparison of public versus private providers because the private sector is very heterogenous ranging from itinerant drug sellers, patent medicine dealers to private teaching hospitals. Also, the public sector is also heterogenous ranging from dispensaries, primary healthcare (PHC) clinics, health posts, PHC centres, comprehensive health centres and hospitals. Hence, many a times, it is not clear the aspect of the private sector and the public sector that are been compared. Therefore, a worthwhile comparison for programmatic purposes is investigating what happens in hospitals versus non-hospitals. Hence, the paper provides information about the characteristics and determinants of provision of malaria treatment by hospitals and non-hospitals, factors that influence provision of malaria treatment services and potential areas for intervention to improve malaria treatment provision.

## Methods

### Study area

The study was undertaken in Anambra state, southeast Nigeria. This part of the country is one of the most important sources of anti-malaria drugs in Africa and the "Bridge-Head" market in Onitsha, Anambra State is at the centre of this trade. Anambra state has a high malaria transmission rate all year and the annual incidence rate is 10 to 35%. Six sites were chosen for the study. These were the three largest urban centres (Awka- state capital, Nnewi and Onitsha). One rural LGA was randomly selected from each senatorial zones in the state (Njikoka, Aguata and Ogbaru). Then, one community from each of the three rural LGAs: Enugwu-Ukwu (Njikoka LGA), Ekwulobia (Aguata LGA) and Okpoko (Ogbaru LGA) was selected using two-stage sampling. Each site area has a full complement of providers from hospitals to itinerant drug providers and herbalists.

### Data collection

It was a cross-sectional study using pre-tested structured questionnaires to collect information from a broad spectrum of healthcare providers by trained interviewers. The questionnaire was administered to the heads or owners of selected public and private providers/outlets. The employee running the facilities/outlets was interviewed in the absence of the owner/head. Data were collected on the level of providers' knowledge regarding malaria, provision of malaria treatment services (diagnosis, treatment and follow-up), and influences on provider behaviour.

#### Sampling and sampling method

The sample size was determined by considerations of the range of providers and feasibility. A total of 50 providers (public and private) in each urban and 25 in each rural area were selected. The breakdown among private providers was determined on the basis of their utilization rate by consumers, using existing data [19]. All the existing public providers in each study area were included in the study, because there are not many of them. Two-stage sampling was used to select the providers so that all categories of public and private providers were well represented in the study. The sampling frame were providers that use orthodox drugs to treat patients and they included all levels of care in public facilities and the broad spectrum of private providers ranging from hospitals, clinics, pharmacy shops, patent medicine dealers and mixed goods dispensers. A listing of all providers was undertaken using the membership registers of their professional associations or trade unions. A "snowball" approach was used to update these initial lists, by asking registered providers to furnish names and locations of others that are not in the registers of their respective associations.

### Data collected

Data was collected on the level of education of providers', their knowledge regarding malaria, their perceptions and practices of provision of malaria treatment services (diagnosis, treatment and follow-up), and influences on provider behaviour.

### Data analysis

The data was analysed for aggregated data from hospitals and non-hospitals using SPSS and EpiInfo software packages. The main influences on provider behaviour were evaluated through quantitative analysis of the provider questionnaire responses. Chi-square tests were used to compare the elicited data between hospitals and non-hospitals.

## Results

### General characteristics of the providers

A total of 137 PMDs, four mixed goods sellers, 22 PHC centres, six maternity homes, 20 private hospitals, three other low level providers, such as community-health workers and itinerant drug sellers, eight laboratories, 11 pharmacy shops, 11 public hospitals and three other types of hospitals were interviewed. Thus, there were 34 hospitals and 191 Non-hospitals. Amongst, the non-hospitals, those in maternity homes had the highest average number of years of formal training for the work that they do, whilst providers in 'others' category had the highest number of formal education. Similarly, it was providers in 'other healthcare facilities' that had the highest number of years of training for the work that they do, whilst providers in private hospitals, the general hospitals had the highest level of education for medium/high level providers.

### Knowledge of the providers about causes, symptoms, diagnosis and treatment of malaria

The three most important illnesses that the providers' clients had were malaria (91.4%), diarrhoea (39.8%) and typhoid fever (32.0%). Most of the providers correctly identified mosquitoes as the cause of malaria and the difference between providers in hospitals and non-hospitals was no statistically significant (Table 1). However, there were more instances of erroneous identification of main causes of malaria by providers in non-hospitals compared to hospitals, where drinking impure water, lack of environmental sanitation and exposure to sunlight were identified as causes of malaria.

**Table 1:** Main causes of malaria

	**Non-hospitals** **n (%)** **N = 191**	**Hospitals** **n (%)** **N = 34**	**Chi-square(p-value)**
Mosquitoes	176 (92.5%)	33 (97)	1.7 (.17)
Lack of sanitation	106 (55.4%)	9 (26.5)	9.1 (.003)
Drinking impure water	32 (16.7%)	2 (5.88)	2.5 (.08)
Exposure to sunlight	15 (7.8%)	0 (0)	2.8 (.08)
Others	42 (21.9)	3 (8.8)	3.0 (.06)

Comparatively, all (100%) providers in hospitals and just 87.4% of non-hospitals stated that malaria was a serious health problem (p < 0.05). The main method that the providers stated that could be used to diagnose malaria was through recognition of symptoms (85.2%). Microscopic examination of blood slides was stated by only 27.3% of the providers. All the providers equally recognized all the symptoms of malaria, with the exception of headache, which was recognized less by hospitals and vomiting, which was recognized more by hospitals (Table 2). Surprisingly, convulsion and anaemia were the least recognized symptoms of malaria by both hospitals and Non-hospitals, although there was far greater recognition of anaemia in hospitals compared to non-hospitals (p < 0.05).

**Table 2:** Symptoms of malaria

	**Non-hospitals** **n (%)** **N = 191**	**Hospitals** **n (%)** **N = 34**	**Chi-square (p-value)**
Fever	173 (90.5%)	33 (97%)	2.2 (.23)
Shivering	63 (33.0%)	15 (44.1%)	1.8 (.24)
Convulsion	9 (4.7%)	3 (8.8%)	1.0 (.39)
Headache	176 (92.5%)	26 (76.5%)	5.3 (.028)
Anaemia	5 (2.6%)	16 (47.1%)	9.8 (.002)
Vomiting	79 (41.4%)	20 (58.8%)	4.0 (.036)
Diarrhoea	11 (5.8%)	1 (3.0%)	.43 (.44)
Loss of appetite	113 (59.2%)	19 (55.8%)	.05 (.48)
Fatigue	81 (42.4%)	14 (41.2%)	.002 (.56)
Others	127 (66.5%)	28 (82.4%)	3.9 (.033)

Most of the providers; 88.2% and 87.4% in hospitals and non-hospitals respectively stated that recognition of symptoms could be used to tell that someone has malaria. However, whilst 58.8% of providers in hospitals stated that microscopy could be used to diagnose malaria, only 30.4% of providers in non-hospitals had a similar opinion (p < 0.05). Only 2 (5.9%) of hospitals and no non-hospital stated that rapid diagnostic tests (RDTs) could be used to diagnose malaria (p < 0.05). 58.8% of the hospitals had functional microscopes, reagents and slides for microscopy. Conversely, only 15.7%, 14.7% and 14.7% of non-hospitals had functional microscopes, reagents and slides. The differences between hospitals and non-hospitals were statistically significant (p < 0.05).

### Practices of the providers

Table 3 shows that in actual practise, malaria diagnosis was based mostly on recognition of symptoms by 82.4% of hospitals and 87.4% of non-hospitals. However, whilst 73.5% of hospitals used microscopy to diagnose malaria, only 34.5.1% of non-hospitals did (p < 0.05). Also, only 5.9% and 0% of hospitals and non-hospitals used RDT (p < 0.05). Incidence of patients using self recognition of symptoms to ask for treatment was found mostly in non-hospitals.

**Table 3:** How malaria is actually diagnosed

	**Non-hospitals** **n (%)** **N = 191**	**Hospitals** **n (%)** **N = 34**	**Chi-square (p-value)**
Physical exam only	52 (27.2)	12 (35.9)	1.3 (.17)
Recognition of symptoms	167 (87.4)	28 (82.4)	0.14 (.54)
Microscopy	66 (34.5)	25 (73.5)	20.5 (.0001)
Dipstick (RDT)	0 (0)	2 (5.9)	11.9 (.020)
Sufferer recognize & asks for treatment	21 (11.0)	7 (20.6)	2.9 (.085)
Others	21 (11.0)	4 (11.8)	.053 (.51)

82.4% of hospitals and 66.7% of non-hospitals stated that taking medicines was the best way to cure malaria (Table 4). However, 1%, 24%, 5.8% and 0.5% of non-hospitals stated that no treatment, changing the diet, cleaning the environment and religious healing could be used to cure malaria.

**Table 4:** Best ways to cure malaria

	**Non-hospitals** **n (%)**	**Hospitals** **N (%)**	**Chi-square (p-value)**
No treatment	2 (1.0)	0 (0)	.34 (.73)
Medicine	126 (66.7)	28 (82.4)	5.27 (.015)
Change diet	46 (24.0)	0 (0)	9.8 (.0001)
Clean house, environment or body	11 (5.8)	0 (0)	2.0 (.17)
Religious healing	1 (0.5)	0 (0)	.17 (.86)
Herbal medicines	0 (0)	0 (0)	NA
Others	53 (27.7)	13 (38.2)	2.1 (.11)

The drugs that all providers stocked for the treatment of malaria included artesunate monotherapy, chloroquine (CQ) tablets, CQ injections, antibiotics, SP, quinine, ACT, herbal preparations, halofantrine and others. Chloroquine and SP were the most common medications that the providers give to their patients. Comparatively, whilst 44.1% of hospitals provided artesunate monotherapy, it was 31.4% for non-hospitals. Also, 11.2% of hospitals and 7.9% of non-hospitals provided ACT to their patients. Quinine was provided by 41.2% of hospitals and 23% of non-hospitals (p < 0.05). Herbal treatment was also provided by 5.9% of hospitals and 1% of non-hospitals.

Table 5 shows that less stock-outs of drugs occurred in hospitals compared to non-hospitals (p < 0.05). Tablets followed by injections were the most common anti-malarials drug formulations provided. Table 6 shows that hospitals tended to give more injections and intravenous infusions to patients compared to non-hospitals (p < 0.05).

**Table 5:** Level of stock-outs of anti-malarial drugs in the past one month

	**Non-hospitals** **N = 191**		**Hospitals** **N = 34**		**Chi-square (p-value)**
	**Yes** **n (%)**	**Don't normally stock** **n (%)**	**Yes** **n (%)**	**Don't normally stock** **n (%)**	

AMT	17	18	2	13	23.0 (.0001)
CQ	111	1	5	1	20.4 (.0001)
Antibiotics	16	5	4	3	4.6 (.10)
SP	97	11	3	6	21.0 (.0001)
Quinine	17	25	5	9	7.5 (.023)
Halofantrine	24	28	3	14	15.9 (.0001)
Others	46	5	5	1	1.0 (.60)

**Table 6:** Types of drug formulations most commonly given to patients for malaria

	**Non-hospitals** **n (%)**	**Hospitals** **n (%)**	**Chi-square (p-value)**
Injection	67 (35.0)	27 (79.4)	27.8 (.00001)
Tablets	177 (92.7)	30 (88.2)	.16 (.51)
IV infusion	15 (7.9)	12 (35.3)	23.0 (.0001)
Suppositories	5 (2.6)	2 (5.9)	1.2 (.26)
Others	41 (21.5)	10 (29.4)	1.6 (.15)

### Factors influencing malaria treatment provision: motives and incentives for service provision

Majority of the providers considered ability to pay bills (35.2%), already existing relationship (9.4%) and body mechanism - cursory ill-health appearance of the patient - (35.2%) of the patient before they provided specific malaria treatment services to the patients. There were no statistical significant differences in consideration of ability to pay bills and already existing customer relationship between hospitals and Non-hospitals. However, 37.2% of non-hospitals and 58.8% of hospitals considered body mechanisms (how the patients visually looks - weak, collapsing or otherwise) before commencing treatment (p < 0.05).

Table 7 shows that pressures from wholesalers to providers to repay the cost of supplied drugs was the major influence of the type of drugs provided to patients and there was no statistically significant differences between hospitals and non-hospitals. Request by patients was the next commonest factor influencing treatment but it was more evident in non-hospitals compared to hospitals (p < 0.05). Also, respondents said that request by the patients, patient's apparent socio-economic status (SES), their idea of what the patients want and drugs available were the major determinants of the amount of money they charge for an episode of malaria. The necessity to make a profit was a more important factor in Non-hospitals compared to hospitals (p < 0.05). Other determinants were level of training of providers, necessity to make profit, need to be competitive, drugs available and existing treatment policy/guidelines. There was no statistical significant difference in factors influencing the amount of money charged for treatment between hospitals and non-hospitals.

**Table 7:** Factors influencing the type of drugs provided for patients

	**Non-hospitals** **n (%)** **N = 191**	**Hospitals** **n (%)** **N = 34**	**Chi-square (p-value)**
Necessity to make profit	18 (9.4)	3 (8.8)	.01 (.60)
Request by patients	76 (39.8)	5 (2.6)	6.7 (.006)
Idea of what the consumer prefers	17 (8.9)	5 (2.6)	1.5 (.19)
Patients apparent SES	37 (19.4)	10 (29.4)	2.4 (.096)
Providers' level of training	31 (16.2)	5 (2.6)	.004 (.594)
Need to be competitive	5 (2.6)	0 (0)	.85 (.46)
Drugs available	35 (18.3)	2 (5.9)	2.8 (.07)
Existing treatment policy/guidelines	26 (13.6)	1 (2.9)	2.7 (.07)
Information from regulatory bodies	10 (5.2)	0 (0)	1.7 (.21)
Pressures from wholesalers to repay	89 (46.6)	22 (64.7)	.17 (.85)
Promotion by manufacturers	3 (1.6)	0 (0)	.50 (.63)
Others	14 (7.3)	12 (35.3)	5.6 (.014)

## Discussion

The level of knowledge about malaria by all the providers was good, although it was worrisome to find out that some providers had the wrong notion about the cause of the diseases and many providers failed to recognize that convulsion and anemia were symptoms of malaria. Some other researchers also found in their study of PMDs, that although the respondents were well informed about causes and symptoms of malaria, there were some misconceptions about the causes of malaria, incriminating the hot sun and drinking bad water [2]. The finding that convulsion was not stated as a symptom of malaria by many of the providers has serious implications for treatment of the disease as many people will not be treated if convulsion is the presentation [2]. However, it was observed that most of the providers stated that malaria was a very serious illness [2].

Laboratory diagnosis can improve rational provision of malaria treatment services, but the results show that only few providers, including hospitals, actually use any laboratory diagnosis. The finding of low use of microscopy for diagnosis in the study differs from the findings of a previous study where there was a high level of use of microscopy [10]. The disparity could be because the other study [10] was based on audit of patients' records from hospitals, whilst the present study was based on data collected from all levels of healthcare providers. Syndromic diagnosis (presumptive treatment) of malaria results in unnecessary prescribing of anti-malarials [20]. It was found that prescribing anti-malarials only after laboratory confirmation reduced the total number of prescriptions by 68% in Malawi [20].

There was apparent irrational provision of anti-malarial drugs in the study and it was interesting to find out that providers stocked the full range of anti-malarials including the failing ones and that some provided antibiotics to their patients as part of malaria treatment. Whilst this may be rational drug provision of antibiotics in some instances, it may be irrational in other cases if the drugs are not properly provided in correct type, dosage and indication. Antibiotics are overused in both rural and urban settings of a district in Tanzania and this was due to both clinician's and drug sellers' prescribing practices in public and private facilities [21]. In many situations, practices that could be best described as misuse of drugs have become routine, and in many cases, institutionalized and promoted [5]. An example is syndromic diagnosis that was widespread in the study area. As was found in this study and other studies [22], injection use is often unnecessary. Similarly it was found that prescribing for adult malaria by many providers did not conform to the treatment guidelines [23].

Many interesting factors that were found to influence providers' malaria treatment provision behaviour are pointers to how such a behaviour could be modulated to improve the treatment of malaria. The finding that patient demand was a significant factor that affected treatment provision of non-hospitals, but not of hospitals, supports the results of earlier studies that found that prescribing patterns are more likely to follow patient demands for patent medicine dealers [2], but that patient demand for anti-malarials would not affect the prescribing behaviour of clinicians in hospitals[14]. The finding that patients' body mechanism on presentation to the providers was a strong determining influence for the commencement of treatment especially in hospitals was not unexpected given that training of doctors and other providers usually found in hospitals emphasize the use of triage in deciding on who and when to start treatment especially in emergencies. The result showed that hospitals were very likely to commence immediate treatment if they notice apparently very ill patients and may not start treatment of the patient does not appear quite ill. It will be important to educate all providers that the body mechanism of clients though important should not overtly outweigh other considerations for starting treatment as some people especially children that may not look quite ill, could actually be dying.

In this study, the finding that pressures from wholesalers to the providers to repay the cost of drugs supplied to them was a major determinant of the drugs provided is worrisome. This is because, it could lead to a lot of supplier-induced demand and provision of unnecessary drugs thereby worsening the economic burden of the disease on the consumers and predisposing to development of drug resistance if the drugs are wrongly used. However, a study of patent medicine dealers in Nigeria showed that customers' specific demand was the main determining factor for the treatment provided [2]. A previous study done to explore to what extent current prescribing behaviour is driven by patient demand in five district hospitals found that patients were not observed to demand anti-malarials from clinicians but occasionally asked for a malaria slide and that clinicians rarely reported perceiving patient demand for anti-malarials asserted that such demand for medication would not affect their prescribing behaviour [14].

Interventions at policy and programmatic levels should be instituted so as to improve treatment provision. One obvious intervention will be ensuring that providers stocked adequate doses of ACT and improving laboratory diagnosis especially as the expensive forms of ACT are now first-line treatment of malaria, so as to decrease unnecessary treatment and reduce societal costs of the disease. Interventions are also needed to decrease the use of injections especially in hospitals, which is a common means of treatment in Nigeria [2]. Other interventions would entail behaviour change and communication to improve providers' behaviour. There have been suggestions that continuing education to prescribers and providers, monitoring, supervision and public education could be used to curb irrational drug prescribing [22]. It has been acknowledged that although changing private sector practices is widely acknowledged to be slow and difficult [24], this change helps to improve their treatment provision practices [25]. Previous studies have shown that vendor-to-vendor education improved the treatment of malaria in private outlets in Kenya, by improving their stocking patterns, malaria knowledge and prescribing practices of shops/kiosks, but not consistently on other types of outlets [24]. Similarly, training of providers, especially drug sellers, has been shown to improve drug use in Kenya [26].

## Conclusion

Finally, there are many challenges to appropriate provision of malaria treatment services, although challenges are less in hospitals compared to other types of healthcare providers. Ensuring adequate supply of appropriate drugs, using favourable drug supply terms that will not pressurize providers to indulge in supplier-induced demand or over-prescription, limiting the influence of patients in demanding specific treatment, improving proper diagnosis of malaria and improving the knowledge of providers about malaria are interventions that could be used to improve malaria treatment provision. However, the desire to limit provision of anti-malarial drugs to those who need them fails to account for the systematic realities that limit the availability of accurate diagnosis to only a small fraction of the population [5]. Hence in the interregnum between widespread use of laboratory diagnosis whether through microscopy or RDTs, the health system should include strategies such as Integrated management for childhood illnesses (IMCI), Integrated preventive treatment for pregnant women (ITPp) and possibly Integrated preventive treatment for infants (IPTi), to ensure that everybody that needs malaria treatment is properly treated, without having to necessarily wait for laboratory confirmation of diagnosis.

## Competing interests

The authors declare that they have no competing interests.

## Authors' contributions

OO conceived and designed the study. All the authors participated in data collection and analysis. OO wrote the first draft and all the authors contributed to and revised this draft until the final draft was produced for publication.
